# The Belgian Doctors and Pharmacists

**Published:** 1918-01-26

**Authors:** 


### THE BELGIAN DOCTORS AND PHARMACISTS.

The sufferings, material and moral, which the war has inflicted on Belgium are largely concealed from us by the fog of war. We cannot possibly know them fully until peace is restored, and even then there will be much that cannot be spoken. But we do know something of the destitution which has fallen upon a large part of the population, and of the bitter wrongs which the country generally has suffered. Not the least worthy of recognition and sympathy are the disasters which have attended members of the medical profession. Many practitioners have been reduced from comfort and comparative affluence to actual poverty and want. Their own homes destroyed, they have had, so far as possible, to continue to minister to the needs of a pauperised people, and time has not lessened the strain and difficulties of their position. In these circumstances it was natural that the profession in this country and in other parts of the Empire should unite for the purpose of sending a message of practical sympathy to their Belgian colleagues, and with medicine pharmacy was becomingly associated. It was this desire which led to the establishment of the Belgian Doctors' and Pharmacists' Relief Fund soon after the outbreak of the war.

The committee in charge of this fund, including representative members of both professions, has now given an account of its stewardship; and, what is more, appeals for help so that it may continue its work. Of the need let it be clearly understood there is not the slightest doubt. The information to hand, indeed, shows that the misery of the country is an increasing quantity, and in it doctors and pharmacists are involved with their fellow countrymen. The position of educated and cultured men with families dependent on their exertions, deprived not only of the usual advantages of their social status, but even of the means of existence, is a particularly painful one, and it is an actual and continuing experience. Hitherto the British committee has been able to send to Belgium a substantial sum each month. But the fund is now exhausted, and unless it is renewed the consequences will be disastrous to many who by its help have been able to secure, not comfort, but the avoidance of cold and hunger. It is impossible to believe that British liberality will cease at this supreme juncture. We have, it is true, our own difficulties and sufferings, but these will surely quicken our sympathies with colleagues on whom a much heavier lot has fallen. What we have done in the past must not have its grace and value lessened by present failure, and we must show that we are with our Belgian *confrères* even to the end. This end, we reasonably hope, cannot be long delayed, and we may be sure that the gratitude and good will of a gallant nation are no light reward. The appeal is one of honour and good conscience, and we readily accept the opportunity to present it to our readers. The fund has been most economically administered, the guarantee that the contributions have reached those for whom they were intended is secure, and the claim is urgent beyond words. All we need add is that the president of the fund is Sir Rickman J. Godlee, Bart., and the hon. treasurer Dr. H. H. Des Vœux, 14 Buckingham Gate, S.W. 1.

